# Dysmenorrhea among female medical students in King Abdulaziz University: Prevalence, Predictors and outcome

**DOI:** 10.12669/pjms.316.8752

**Published:** 2015

**Authors:** Nahla Khamis Ibrahim, Manar Saleh AlGhamdi, Alanoud Nawaf Al-Shaibani, Fatima Ali AlAmri, Huda Abdulrahman Alharbi, Arwa Kheder Al-Jadani, Raghad Ahmed Alfaidi

**Affiliations:** 1 Nahla Khamis Ibrahim, Professor, Family & Community Medicine Dept., Faculty of Medicine, King Abdulaziz University, Jeddah, Saudi Arabia. Epidemiology Department, High Institute of Public Health, Alexandria University, Alexandria, Egypt; 2 Manar Saleh AlGhamdi, Fifth Year Medical Students, Faculty of Medicine, King Abdulaziz University, Jeddah, Saudi Arabia; 3 Alanoud Nawaf Al-Shaibani, Fifth Year Medical Students, Faculty of Medicine, King Abdulaziz University, Jeddah, Saudi Arabia; 4 Fatima Ali AlAmri, Fifth Year Medical Students, Faculty of Medicine, King Abdulaziz University, Jeddah, Saudi Arabia; 5 Huda Abdulrahman Alharbi, Fifth Year Medical Students, Faculty of Medicine, King Abdulaziz University, Jeddah, Saudi Arabia; 6 Arwa Kheder Al-Jadani, Fifth Year Medical Students, Faculty of Medicine, King Abdulaziz University, Jeddah, Saudi Arabia; 7 Raghad Ahmed Alfaidi, Fifth Year Medical Students, Faculty of Medicine, King Abdulaziz University, Jeddah, Saudi Arabia

**Keywords:** Dysmenorrhea, Prevalence, Predictors, Outcome and Visual Analogue Scale

## Abstract

**Objective:**

To determine the prevalence, predictors and outcome of dysmenorrhea among female medical students in King Abdulaziz University (KAU), Jeddah, Saudi Arabia.

**Methods:**

A cross-sectional study was conducted among 435 medical students at KAU, Jeddah selected through stratified random sample method. A pre-constructed, validated, self-administered questionnaire was used to collect personal and socio-demographic information. Data about menstrual history, stress, smoking were also collected. The severity of dysmenorrhea was scored by the “Visual Analogue Scale (VAS)”. Descriptive and analytical statistics were conducted.

**Results:**

The prevalence of dysmenorrhea was 60.9%. Logistic regression showed that heavy period was the first predictor of dysmenorrhea (aOR=1.94; 95% CI: 1.29- 2.91), followed by stress (aOR=1.90; 95% C.I.: 1.19-3.07). The prevalence of severe dysmenorrhea among the sufferers was 38.6%. Depressed mood was the commonest (80.8%) symptom accompanying dysmenorrhea. Regarding the outcome of dysmenorrhea, 67.5% of the sufferes reported emotional instability, while 28.3% reported absenteeism from the university.

**Conclusions:**

A high prevalence of dysmenorrhea was prevalent among medical students in King Abdulaziz University (KAU), Health promotion, screening programs, and stress management courses are recommended.

## INTRODUCTION

Menstrual disorder is an important health problem among females during their reproductive age.^1^ Dysmenorrhea is defined as a cramping pain that is located in the lower abdomen. It may be associated with headache, dizziness, diarrhea, nausea and vomiting, backache and leg pain.^2^ It is one of the commonest complaints and abnormality among females at their reproductive age.^3^,^4^ It was estimated that about 25-95% of females are complaining from dysmenorrheal.^5^-^7^ It is also associated with greater psychological, physical, behavioral and social distresses.^5^

Morbidity due to dysmenorrhea has a significant impact on public health. It is one of the leading causes of school and work absenteeism and is responsible for greater loss of income and decreased quality of life. Despite its high prevalence and the associated negative effects, many women do not seek medical care for this condition.^6^

A cross-sectional study conducted among medical sciences students from Nigeria, 2012, found that the prevalence of dysmenorrhea was very high among the medical students and it is related to obesity, socioeconomic level, exercise and dietary habits. They also reported that changes in menstrual pattern may affect their physical and mental health.^8^ Although dysmenorrhea represents a big public health problem among females, however, few studies have been conducted about this important problem among medical students in Jeddah.

The objective of the study was to determine the prevalence, predictors and outcome of dysmenorrhea among female medical students in King Abdulaziz University, Jeddah, Saudi Arabia.

## METHODS

This cross-sectional study was conducted at King Abdulaziz University (KAU), Jeddah after administrative approvals. The study population included female medical students who had completed the freshman year (2^nd^ – 6^th^), during the academic year 2013- 2014. A stratified random sample method was used; taking into consideration student’s grade. The sample size was calculated by the following formula:

n= (Z^2^× p× q)/d^2^

n=the minimum sample size, Z= 1.96. The prevalence of dysmenorrhea was assumed to be 35%.^99^ So, p = 0.35 and q =1- p=0.65. The minimum calculated sample size with 5% error and 95% confidence interval = 350. For stratification purposes, the sample size was increased to reach 435 during the fieldwork.

A pre-designed, anonymous, self-administered questionnaire was used. Face and content validity were evaluated by two experts. Reliability was assessed with Cronbach’s α and was found to be 0.83. The questionnaire asked about personal, socio-demographic information and family history of dysmenorrhea. Menstrual history of amenorrhea, amount of blood loss (the number of changed towels /day during menses), and premenstrual symptoms were also asked. Dysmenorrhea was determined by the “presence of two or more days of menstrual pain during menstrual bleeding”. If there was dysmenorrhoea, females were asked about the location (s) of pain.^2^ The questionnaire included the “Visual Analog Scale for pain (VAS)” which is measured on 10 cm scale that representing no pain at one end (0) and the severest imaginable pain written at the other end (10).^10^

### Data analysis

Data was analyzed using SPSS, version 21. According to VAS, there are three ranges of pain: 1–4 for mild pain, 5–6 for moderate pain, and 7–10 for severe pain”.^10^

Chi-squared/Fisher’s exact test, odds Ratios (ORs) and 95% Confidence Interval (CI) were calculated. A logistic regression model was performed to detect the predictors of dysmenorrhea after controlling for confounding factors. Significance was considered at p < 0.05.

Ethical approval was obtained from the Institutional Review Board (IRB) of the Faculty of Medicine, KAU (Reference Number: 134-15) and conformed to the ethical standards of the Helsinki Declaration.

## RESULTS

The study enrolled 435 female medical students with a mean of 21.40±1.4 years. The majority of them were single (94.5%), living with their families (88%). The prevalence of premenstrual symptoms, irregular periods, and secondary amenorrhea were 82.3%, 22.8% and 0.9%, respectively.

The prevalence of dysmenorrhea among medical students was 60.9%, but only 3.2% asked for medical advice. Among the students suffered from dysmenorrhea, 30.6%, 30.8% and 38.6% suffered from mild, moderate and severe pain, respectively. Furthermore, the prevalence of severe dysmenorrhea among the whole female sample was 29.0%.

The prevalence of dysmenorrhea was significantly higher among students enrolled in the basic years compared to those in the clinical years (OR= 1.52; 95% CI: 1.03 -2.65). Table-1. The rate of dysmenorrhea was higher among students with non- enough family income compared to others (*p* < 0.05).

**Table-I:** Relationship between dysmenorrhea and personal, socio-demographic characteristics of female medical students in King Abdulaziz University.

Dysmenorrhea	Yes		No		X^2^	P	OR	95% C.I.
Variable	No.	%	No.	%				
*Age*								
≤20	243	62.0	149	38.0	1.91	0.167	1.56	0.83-2.93
>20	22	51.2	21	48.8				
*Academic year:*								
Basic	176	64.7	96	35.3	4.37	0.037	1.52	1.03-.27
Clinical	89	54.6	74	45.4				
*Marital status:*								
Single	252	61.3	159	38.7	0.49	0.485	1.34	0.59-3.07
Married	13	54.2	11	45.8				
*Father education*								
< University	83	61	53	39	0.001	0.975	0.99	0.66-1.51
University & above	182	60.9	117	39.1				
*Mother’sEducation*								
< university	107	62.2	65	37.8	0.19	0.656	0.91	0.62-1.36
University & above	158	60.1	105	39.9				
*Father occupation*								
Professional	179	60.1	119	39.9	0.29	0.591	0.89	0.59-1.35
Non- professional	86	62.8	51	37.2				
*Mother’s occupation*								
Professional	101	58.7	71	41.3	0.58	0.44	0.85	0.58-1.27
Non professional	164	62.4	99	37.6				
*Residency:*								
With family	236	61.6	147	38.4	0.66	0.41	1.27	0.71-2.29
Dormitory	29	55.8	23	44.2				
*Income*								
Not enough	11	91.7	1	8.3	4.9*	0.02	7.32	1.01-57.2
Enough or exceed	254	60.0	169	40.0				

* Fisher’s exacts test

Students who had stress suffered from dysmenorrhea (64.3%) more than the others (48.3%). Table-II. A highly statistical significant difference was present (*X^2^* = 7.80, *p* < 0.01). Furthermore, the prevalence of dysmenorrhea was significantly higher among students who had family history of dysmenorrhoea. Females who complained from heavy periods (> 3 towels / day) were about 2 times (OR=1.90; 95% CI: 1.27-2.83) more prone to develop dysmenorrhea compared to others. Smokers and those who didn’t practice physical activity had higher prevalence of dysmenorrhea compared to others. However, no statistical significance differences were found (*p*>0.05).

**Table-II:** Relationship between dysmenorrhea anddifferent characteristics of female medical students in King Abdulaziz University.

Dysmenorrhea	Yes		No		X^2^	P	OR	95% CI
Variable	No.	%	No.	%				
Smoking*								
Yes	8	80.0	2	20.0	1.56*	0.21	2.62	0.55-12.46
No	257	60.5	168	39.5				
*Practice physical exercise*								
Yes	95	57.2	71	42.8	1.54	0.21	0.78	0.53-1.16
No	170	63.2	99	36.8				
*Stress*								
Yes	220	64.3	122	35.7	7.80	0.005	1.92	1.21-3.06
No	45	48.4	48	51.6				
*Age of menarche*								
≥15	26	53.1	23	46.9	1.43	0.23	0.70	0.38-1.26
<15	239	61.9	147	38.1				
*Duration between menstrual cycle*								
£ 35	235	59.8	158	40.2	2.16	0.14	0.60	0.30-1.20
³ 35	30	71.4	12	28.6				
*Duration of bleeding*								
£ 5 days	53	53.5	46	46.5	2.93	0.08	0.67	0.43-1.06
> 5 days	212	63.1	212	36.9				
*Amount of bleeding*								
> 3 towels / day	128	69.6	56	30.4	10.01	0.002	1.90	1.27-2.83
≤ 3 towels / day	56	61.2	37	38.8				
*BMI*^•^								
Normal weight	190	63.0	113	37.0	0.354	0.84	1.12	0.70-1.81
Over & obese	56	61.2	37	38.8				
*Family history of dysmenorrhoea*								
Yes	124	66.7	62	33.3	4.51	0.03	1.532	1.03-2.27
No	141	56.6	108	43.4				

• N.B. 37 cases didn’t report her weight and/or height

* Fisher’s exacts test

After controlling confounding factors in the logistic regression model, heavy period was the first predictor of dysmenorrhea (aOR=1.94; 95% CI: 1.29- 2.91), followed by presence of stress (aOR=1.90; 95% C.I.: 1.19-3.07). Table-III.

**Table-III:** Logistic regression analysis of predictors of dysmenorrhea among medical students in King Abdulaziz University.

Predictor	B	Sig.	aOR	95% C.I.
Heavy period	0.663	0.001	1.94	1.29- 2.91
Stress	0.647	0.007	1.90	1.19- 3.07
Constant	- 2.883	0.000		

aOR: “Adjusted Odds Ratio” C.I.: “Confidence Interval”

Depressed mood was the commonest (80.8%) symptom accompanying dysmenorrhea, followed by anger (78.5%). Table-IV. Eating more than usual (46.0%), headache (45.7%), nausea (42.3%), dizziness (38.1%), diarrhea (33.6%) and constipation (22.3%) were the main symptoms.

**Table-IV:** Symptoms accompanying dysmenorrhea among female medical students in King Abdulaziz University.

Symptoms	%
Depressed mood	80.8
Anger	78.5
Eating more than usual	46.0
Headache	45.7
Nausea	42.3
Dizziness	38.1
Diarrhea	33.6
Constipation	22.3
Vomiting	18.5
Phobia	14.7
Syncope	5.7

NB: Every question was separately asked.

The different outcomes of dysmenorrhea among the complainers are shown in Table-V.. Emotional instability was the commonest (67.5%) outcome of dysmenorrhoea, followed by limited daily activity (54.7%), sleep disturbance (54.0%), reduced concentration (50.9%), decreased social activities (50.6%) and absenteeism from university (28.3%).

**Table-V:** Outcomes of dysmenorrhea reported by female medical students in King Abdulaziz University.

Symptoms	%
Emotional instability	67.5
Limited daily activity	54.7
Sleep distrubance	54.0
Reduced concentration	50.9
Decreased social activities	50.6
Absenteeism from university	28.3

NB: Every question was separately asked.

## DISCUSSION

In this study the prevalence of dysmenorrhea was 60.9%, which is slightly higher than the rate reported from Turkey (55.5%).^11^ On the other hand, there is a big global variation in the prevalence of the dysmenorrhea. It was found to be 72.4% among university students from India,^5^ 70% from Italy,^12^ 80% among females aged 15–17-year-old from Australia.^13^ These variations may be due to differences between the target populations, lifestyle, or due absence of a standardized universally accepted method for defining dysmenorrheal.^13^,^14^

It was reported that 21.7% of the Iranian women aged 16 - 56 years suffered from severe pain. The current study reported a higher prevalence by VAS (29.0% among the total sample, and 38.6% among the sufferers from dysmenorrhea). The causes of discrepancies may be attributed to younger age of participants in the current study or due to different used scales.^15^

In the current study heavy period was the first predictor of dysmenorrhea. Similarly a study from Athens, Greece, found that there was a good correlation between heavy and painful periods.^3^

Stress was one of the predictors of dysmenorrhea in the current study, after controlling confounding. Similar results were reported by other studies.^15^,^16^ Several studies give an explanation for this association by relating stress with the cascade of neuro-endocrine responses^15^ Dorn, et al.^17^ reported that emotional disturbance may cause menstrual cycle abnormalities, especially dysmenorrhea which agrees with our results. A statistical association was found between family history of dysmenorrhea and presence of dysmenorrhea among students. which agrees with results from Dammam,^9^ and Iran.^15^

Our results showed that the prevalence of dysmenorrhea was higher among smokers compared to non-smokers (with no statistical significant difference). This agrees with the results of the study from Dammam Saudi Arabia.^9^ On the other hand, these results disagree with results from Turkey^11^ and this may be because the rate of smoking among females in the current study is lower than that from Turkey.

Our study showed that there is no association between BMI and dysmenorrhea which disagrees with other studies.^16^ (12This discrepancy may be due to differences between the study populations. Results of the present study found that there is no association between age of menarche and dysmenorrhea, which concurs with the results from Dammam.^9^ However, these results disagree with results from Egypt.^2^

The prevalence of premenstrual symptoms was 82.3% in the current study, which coincides with studies from Jordan^18^ and Spain.^19^ On the other hand, Tabassum, et al.^20^ from Pakistan reported a lower rate (53%).

As regards the outcomes of dysmenorrhea, Hillen, et al.^13^ found that dysmenorrhea limited the activities of 53% of young Western Australian girls suffered from it which agrees with results of our study. Furthermore, our study showed the rate of absenteeism due to dysmenorrhea was 28.3%, which agrees with results of an older study of Bergsjø, et al.^21^ On the other hand, this rate is much lower than the rate reported among adolescent girls in USA,^22^ which may be attributed to the differences between the two target populations.

Only 3.2% of students in the current study sought medical advice for dysmenorrhea which is much lower than the rate reported from Australia (18%) (21). This may be because females in the current study may believe that the painful periods are normal with no need to seek medical advice, or may be because of their shyness to go to gynecologist.

## CONCLUSIONS

A high prevalence of dysmenorrhea was prevalent from the current study. The main predictors were heavy peoid and stress. This problem requires attention through conduction of health promotion and screening programs. Stress management courses for minimizing possible consequences and outcomes of dysmenorrhea among medical students.
